# Information Wanted

**Published:** 1882-04-20

**Authors:** A. G. Smythe

**Affiliations:** Baldwyn, Miss.


### INFORMATION WANTED.

*Messrs. Editors Southern Medical Record:*

I wish to ask one or two questions, and will be pleased to receive replies from any source capable of answering them.

A neighboring practitioner, waiting upon a woman, in labor, two children were born in due course of time, it is supposed: One of which it is said did not take the proper color, (*said to have been of a purplish color*,) cyanosed I suppose, was cold and did not breathe well.

The attending physician is said to have given calomel; the child survived but a few hours, probably six or eight, not twenty-four as I am informed. Without going into any detailed history of the case, the supposition is that it was true cyanosis. If so, what was the object in giving the calomel?

In another case, a would-be surgeon was consulted or called to see a case of false ankylosis of the knee, after rather long confinement in the treatment of a fractured femur, in a hysterical habit. It was in early winter, the patient occupied a close, warm room, doors and windows closed all the time, large open fireplace, with good wood fires, the patient on a good mattress or bed, generally covered with from ten to twelve quilts, blankets and coverlets; the whole time being enveloped with no less than two, and from that to four doubles of moderately thick flannels. About the first direction was to enclose the knee with several bats of wool in addition to what has been already named. And for some time that was probably all he did. Taking the case as stated, what benefit was likely to occur, or what was the object of the treatment?

These cases are not hypothetical, and are not offered for amusement, but to elicit candid replies, and further to show that the ignorant midwife, who extracted a dead fetus from a suffering woman, with a pair of smith's tongs, when there was no other assistance within reach, is not to be held up to scorn and obloquy, whilst the country is overrun by a class of men, who are supported and forced to the front rank of the profession, to the exclusion of worthy and meritorious members, who will not condescend to pander to the whims, prejudices, and vices of the ignorant and malicious; but sacrifice their popularity, both professional and personal, to say nothing of their pecuniary interest, to maintain the standing of the profession. A. G. SMYTHE.

*Baldwyn, Miss., 20th March, 1882.*

